# The Value of Multidisciplinary Team Meetings for Patients with Gastrointestinal Malignancies: A Systematic Review

**DOI:** 10.1245/s10434-017-5833-3

**Published:** 2017-03-23

**Authors:** Yara L. Basta, Sifra Bolle, Paul Fockens, Kristien M. A. J. Tytgat

**Affiliations:** 1 Department of Gastroenterology and Hepatology, Academic Medical Center, University of Amsterdam, Amsterdam, The Netherlands; 2 Faculty of Social and Behavioral Sciences, University of Amsterdam, Amsterdam, The Netherlands

## Abstract

**Introduction:**

The incidence of gastrointestinal (GI) cancer is rising and most patients with GI malignancies are discussed by a multidisciplinary team (MDT). We performed a systematic review to assess whether MDTs for patients with GI malignancies can correctly change diagnosis, tumor stage and subsequent treatment plan, and whether the treatment plan was implemented.

**Methods:**

We performed a systematic review according to the Preferred Reporting Items for Systematic Reviews and Meta-Analyses guidelines. We conducted a search of the PubMed, MEDLINE and EMBASE electronic databases, and included studies relating to adults with a GI malignancy discussed by an MDT prior to the start of treatment which described a change of initial diagnosis, stage or treatment plan. Two researchers independently evaluated all retrieved titles and abstracts from the abovementioned databases.

**Results:**

Overall, 16 studies were included; the study quality was rated as fair. Four studies reported that MDTs changed the diagnoses formulated by individual physicians in 18.4–26.9% of evaluated cases; two studies reported that MDTs formulated an accurate diagnosis in 89 and 93.5% of evaluated cases, respectively; nine studies described that the treatment plan was altered in 23.0–41.7% of evaluated cases; and four studies found that MDT decisions were implemented in 90–100% of evaluated cases. The reasons for altering a treatment plan included the patient’s wishes, and comorbidities.

**Conclusions:**

MDT meetings for patients with a GI malignancy are responsible for changes in diagnoses and management in a significant number of patients. Treatment plans formulated by MDTs are implemented in 90–100% of discussed patients. All patients with a GI malignancy should be discussed by an MDT.

**Electronic supplementary material:**

The online version of this article (doi:10.1245/s10434-017-5833-3) contains supplementary material, which is available to authorized users.

The incidence of gastrointestinal (GI) malignancies is rising, and, in The Netherlands, the incidence of colorectal carcinoma is expected to increase by 40% in 2020.^1^ To ensure the best care for every patient, a multidisciplinary approach seems important. The multidisciplinary approach to patients with cancer has been described in the literature since 1975^2^; however, multidisciplinary teams (MDTs) have only been in effect since the late 1990s. Since MDTs likely require an increase in available time and recourses, their existence has been questioned.^3^,^4^

In the last two decades, this multidisciplinary approach, often implemented as MDTs, has become routine in our healthcare system. An MDT consists of healthcare specialists from different medical specialties working together for specific diseases.^5^^–^ 7 These teams meet at periodic intervals (i.e., daily or weekly) to discuss and diagnose patients with complex diseases such as cancer, and subsequently formulate a treatment plan according to the current guidelines.^5^^–^ 8 In this systematic review, this will be referred to as the MDT meeting (MDTM). Several articles relating to various oncological diagnoses show valuable insights into the effectiveness of MDTMs.^5^,^8^^–^ 14 For instance, patients discussed in an MDTM have been shown to be more satisfied and more often receive a correct diagnosis and treatment plan according to guidelines.^5^,^9^^–^ 14 Although this suggests that MDTs are able to improve patient care, improved patient outcomes, such as survival, have not yet been established.^8^

Patients with GI malignancies comprise a large portion of all cancer patients. Often, gastroenterologists and surgeons specialized in GI malignancies are interested in more than one tumor type. Therefore, we felt it was important to evaluate patients with GI malignancies as one group. The aim of this systematic review was to assess whether there is scientific evidence in the literature that discussion in a multidisciplinary GI cancer team meeting influences the diagnosis and treatment plan for patients with GI malignancies. We specifically focused on whether an MDT can correctly change diagnosis and tumor stage. Additionally, this review aimed to evaluate whether the subsequent treatment plan was also changed and whether a treatment plan formulated by an MDT was implemented.

## Methods

This systematic review was performed according to the Preferred Reporting Items for Systematic Reviews and Meta-Analyses (PRISMA) guidelines.^15^

### Search Strategy and Eligibility Criteria

A systematic literature search was performed in the PubMed, MEDLINE and EMBASE electronic databases. The free text and Medical Subject Heading search terms used were variations of ‘multidisciplinary’ and ‘team’, ‘correct diagnosis’, ‘changes in diagnosis’, ‘survival’, ‘guidelines adherence’, and ‘gastrointestinal neoplasm’ (the detailed search is documented in Appendix). No language or time restrictions were set, and references of lists of the included articles were hand searched. The last search was run on 30 November 2016.

Articles were included when the following criteria were fulfilled: studies that relate to adults with a GI malignancy and describe a change in initial diagnosis, stage, or treatment. All study types were included, with the exception of case series due to the high potential bias in this study design. Review articles were assessed to ensure the articles evaluated were found in our search. Thereafter, review articles were excluded. Two researchers (YB and SB) individually applied these criteria to all retrieved titles and abstracts. Any disagreements were first discussed between the two researchers and, if no agreement could be reached, an independent investigator with expert knowledge of the field was contacted (KT). Reference lists of included articles were screened to ensure no relevant articles had been missed.

### Data Extraction

A data extraction sheet was developed for this study, based on information provided by the Centre for Reviews and Dissemination.^16^ Two researchers (YB and SB) independently extracted the data, and disagreement was resolved by discussion between the two researchers. Due to the diversity of the data, summary measures or meta-analyses were not appropriate; instead, a narrative description of the findings is reported.

### Quality Assessment

Quality assessment of the included papers was performed with two separate tools created and validated by the National Institutes of Health (NIH). Before–after studies were assessed using the ‘Quality Assessment Tool for Before–After (Pre–Post) Studies With No Control Group’.^17^ With this tool, 12 different criteria could be evaluated and rated with ‘yes’, ‘no’, ‘could not determine’ (CD), and ‘not applicable’ (NA). Cohort studies were evaluated using the ‘Quality Assessment Tool for Observational Cohort and Cross-Sectional Studies’.^17^ This tool had a 14-criteria checklist and could be rated identically to the before–after studies. Two researchers (YB and SB), independently of each other, classified the included papers as ‘poor’, ‘fair’, or ‘good’ using the aforementioned tools. Any disagreement was initially discussed between these two researchers and, if no agreement could be reached, a third reviewer was contacted to make the decision (KT).

The same two researches (YB and SB) also independently assessed the risk of bias and, if no agreement could be reached, the third reviewer was consulted (KT). The risk of bias was assessed for both cohort and before–after studies using a single tool ‘To Assess the Risk of Bias in Cohort Studies’, validated by the Cochrane Institute. With this tool, eight criteria could be rated on a four-item scale: definitely yes (low risk of bias), probably yes, probably no, and definitely no (high risk of bias). Bias was assessed at outcome level.

## Results

**Fig. 1:**
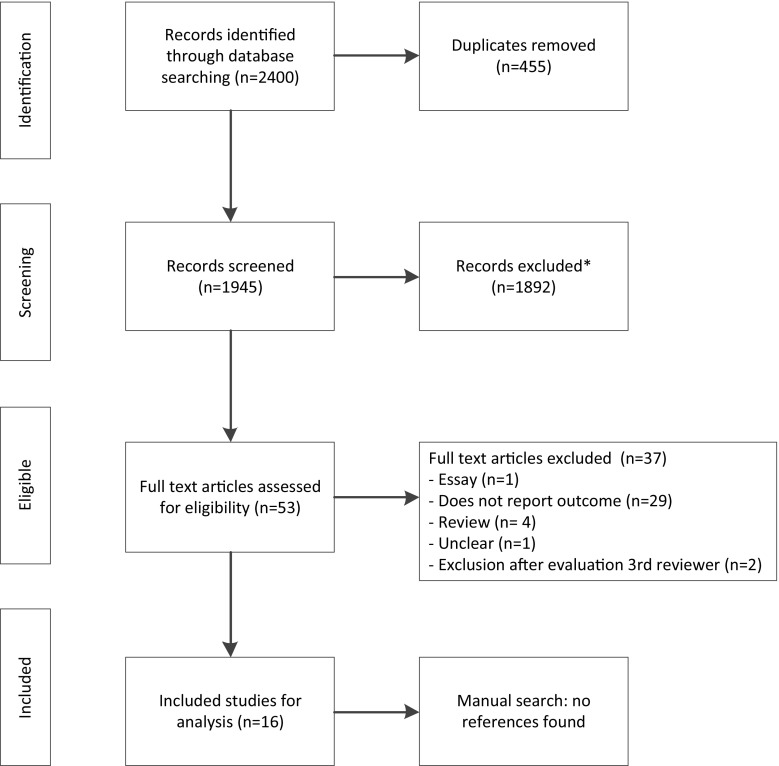
Literature search according to the PRISMA guidelines. ^1^Excluded because titles and abstracts did not meet the inclusion criteria. *PRISMA* Preferred Reporting Items for Systematic Reviews and Meta-Analyses

A total of 2400 articles were retrieved (Fig. 1). The titles and abstracts of these articles were screened to evaluate whether the inclusion criteria were met. Sixteen studies met the inclusion criteria and were the basis for this systematic review.^13^,^18^^–^ 32 Screening the references of the included studies produced no new articles. Study characteristics are described in Table 1. In total, 8018 patients were discussed in 16 articles. Of these patients, 48.5% had an esophageal or gastric malignancy, 25.6% had a colorectal malignancy, 16.3% had a pancreatic or biliary malignancy, 9.1% had a liver malignancy or neuroendocrine tumor, and 0.5% had other malignancies.

**Table 1:** Characteristics of the included studies

Author, year	Inclusion period	Countries	Study designs	Aims	Tumor	Participants	Results
Basta et al., 2016^31^	2012–2013 and 2013	Netherlands	Prospective cohort study	To evaluate the decision-making process of a GI cancer MDT, together with factors influencing this process	HCC, colorectal cancer, esophageal and gastric cancer, biliary and pancreatic cancer	551	21.8% Change in referral dx, of which both stage and dx were changed for 3.2%, stage alone was changed for 4.9%, and dx alone was changed for 12.2%. 6% were diagnosed with benign disease. Different management was advised in 5.8%
Bumm et al., 2007^18^	1999–2006	Germany	Before–after study	To describe the design and operation of a daily intradisciplinary tumor board in a university hospital setting	Gastroesophageal cancer	2450, of which 1545 MDT decisions were evaluated	In 15 and 21% of cases, the MDT rejects modifies the concept decision, respectively. 96% of MDT decisions were implemented
Burton et al., 2006^19^	1999–2002	UK	Retrospective cohort study	To assess the impact of an MDT on implementing an MRI-based preoperative treatment strategy	Rectal cancer	298	For patients discussed by the MDT, the CRM+ rate was 8 versus 26% CRM+ rate for patients NOT discussed by the MDT (*p* < 0.001)
Davies et al., 2006^13^	1999–2002	UK	Prospective cohort study	To investigate the influence of an MDT on clinical staging accuracies and treatment selection	Gastroesophageal cancer	118	The MDT formulates a correct dx in 88–89% of all cases presented, compared with pathological dx
Dickinson et al., 2007^20^	1995–2005 2005–2006	UK	Before–after study	To determine if the introduction of MDT meetings has affected the natural history of this disease	Pancreatic body cancer	Pre-MDT: 23 Post-MDT: 8	More patients received chemotherapy (according to guidelines) post-MDT, 43.5 versus 25.0% pre-MDT (*p* = 0.433). No influence on survival (*p* = 0.376)
Fernando et al., 2015^30^	2013–2014	New Zealand	Prospective cohort study	To determine which patients benefit most from MDTs	CRC	MDT group: 459 Nondiscussed group: 182	An initial management plan was determined in 94 patients, which was changed in 22 (23%) patients after discussion by the MDT. The MDT changed the clinical staging in 20 (4%) cases. Patients with colon cancers are less often discussed in an MDT compared with patients with rectal cancer
Freeman et al., 2011^25^	2001–2004 2005–2007	US	Before–after study	To compare patients with esophageal cancer treated before and after the establishment of a multidisciplinary care conference	Esophageal cancer	Pre-MDT: 117 Post-MDT: 138	97% of patients received a complete staging versus 67% pre-MDT (*p* < 0.0001). In the post-MDT group, 9% endoscopic resection versus 3% pre-MDT (*p* = 0.036)
Meguid et al., 2016^32^	2015	US	Prospective cohort study	To determine if implementation of disease-specific multidisciplinary programs with associated conferences and clinics result in a change of dx and/or change in management for patients	Pancreas and biliary cancer; esophagus and gastric cancer; liver and NET cancer; colorectal cancer	1747	26.9% Change in dx, 20.5% radiographic or endoscopic, resulting in stage change, 4.9% radiographic evaluations that resulted in change in clinical dx, 1.9% change in path review, 6.4% incidental findings, and 28.1% change in treatment recommendation
Oxenberg et al., 2015^21^	2012–2013	US	Prospective cohort study	To assess change in treatment plan from pre- and post-MDT discussion	GI malignancy	Upper GI: 115 Lower GI: 34	36% of initial management plans were changed by the MDT, of which the original stated plan was preceded by additional treatment for 15, and the change was ‘major’ for 38
Pawlik et al., 2008^22^	2006–2007	US	Before–after study	To evaluate the impact of an MDT on the advice of patients compared with prior advice	Pancreatic cancer	203	dx for 38 patients was altered by MDT: 3 patients turned out to be irresectable, 26 were metastasized, 4 patients had benign diseases, and 5 turned out to be resectable
Schmidt et al., 2015^29^	2010–2012	US	Prospective cohort study	To prospectively analyze the evolution in staging and treatment plans and subsequent level of adherence	Esophageal cancer	185	Primary care provider treatment plans were changed for 48 (26%) patients. Diagnostic procedures (staging) were altered for 30 patients (16%). 98% of MDT decisions were followed
Snelgrove et al., 2015^28^	2012–2013	Canada	Prospective cohort study	To assess the quality of the MDT, the effect of the MDT on the original treatment plan, compliance with the MDT treatment plan, and the clinical outcomes	Rectal cancer	42	A change in treatment plan occurred in 29% (*n* = 12) of patients, of which five had their treatment changed because of reinterpretation of the MRI, and six because of tumor factors. One patient had his treatment changed because of comorbidities. All MDT decisions were implemented
van Hagen et al., 2013^23^	NR, duration 8 months	Netherlands	Prospective cohort study	To determine the effect of an MDT on clinical decision making	Upper GI	171	34.5% (*n* = 87) of initial treatment plans changed after discussion by the MDT; 8 changed from curative to palliative, and 2 changed from palliative to curative. For 31, a different treatment modality was preferred, and, for 29, a more extensive workup was needed. For two cases, a different treatment within the same treatment modality was advised
Wood et al., 2008^26^	2005–2006	UK	Prospective cohort study	To analyze if MDT decisions are implemented and what factors influence this	Colorectal cancer	201 Treatment decisions for 157 patients	Of the 20 decisions (10%) that changed after the meeting, the most common reason was comorbidity (*n* = 16). Seven decisions changed due to patient wishes and two changed in light of new clinical information. One was changed by the treating physician
Ye et al., 2012^27^	1999–2006	China	Retrospective cohort study	To assess the effect of MDTs on the management of patients	Colorectal cancer	Pre-MDT: 297 Post-MDT: 298	Pre-MDT, 41.1% of patients underwent CT staging versus 81.3% post-MDT (*p* < 0.001). In the pre-MDT group, 26.7% had liver metastasis 6 months after dx versus 9.3% post-MDT (*p* < 0.05). MDTs increased 5-year survival from 62.4 to 79.1% (*p* = 0.015)
Zhang et al., 2013^24^	2009–2012	US	Retrospective cohort study	To examine how a single-day MDLC affected recommendations compared with prior recommendations	Liver cancer	343	For 26 patients, diagnoses were altered, 8 from malignant/indeterminate to benign, 5 from benign to malignant. Management plans were initially formulated for 168 patients, of which 70 were changed; from irresectable to resectable for 5 patients and vice versa for 4 patients

*MDT* multidisciplinary team, *NR* not reported, *MDLC* multidisciplinary liver clinic, *GI* gastrointestinal, *MRI* magnetic resonance imaging, *HCC* hepatocellular carcinoma, *CRC* colorectal carcinoma, *NET* neuroendocrine tumor, *dx* diagnosis, *CRM* circumferential resection margin, *CT* computed tomography, *Major change* changes between liver-directed therapies, chemotherapy, radiation therapy, type of surgery, ablative therapies, observation and endoscopic procedures^21^

### Quality Assessment

All cohort articles and before–after studies were classified as fair, as evaluated using the quality assessment tools of the NIH (Tables 2, 3). All studies scored a moderate risk of bias, using the bias assessment tool validated by the Cochrane Institute (Table 4).

**Table 2:** Quality assessment cohort studies (NIH)

	Basta et al.^31^	Burton et al.^19^	Davies et al.^13^	Fernando et al.^30^	Meguid et al.^32^	Oxenberg et al.^21^	van Hagen et al.^23^	Schmidt et al.^29^	Snelgrove et al.^28^	Wood et al.^26^	Ye et al.^27^	Zhang et al.^24^
Clear aim	Yes	Yes	Yes	Yes	Yes	Yes	Yes	No	Yes	Yes	No	Yes
Population defined	Yes	Yes	Yes	Yes	Yes	NR	Yes	Yes	Yes	Yes	Yes	Yes
Participation rate of >50%	Yes	Yes	Yes	Yes	Yes	No	Yes	Yes	No	Yes	Yes	Yes
Subjects recruited from the same population	Yes	Yes	Yes	Yes	Yes	Yes	Yes	Yes	Yes	Yes	Yes	Yes
Sample size justification	NR	NR	NR	NR	NR	NR	NR	NR	NR	NR	NR	NR
Exposures of interest measured prior to outcome	No	No	No	No	No	NA	No	No	No	Yes	Yes	No
Sufficient time frame	Yes	Yes	Yes	Yes	Yes	Yes	Yes	Yes	Yes	Yes	Yes	Yes
Different levels of exposure examined	NA	NA	NA	NA	NA	NA	NA	NA	NA	NA	NA	NA
Exposure measures defined	Yes	Yes	Yes	Yes	Yes	Yes	Yes	Yes	Yes	Yes	Yes	Yes
Exposures assessed >1	Yes	No	No	No	No	No	No	No	No	No	No	No
Outcome measures clear	Yes	Yes	Yes	Yes	Yes	Yes	Yes	Yes	Yes	Yes	No	Yes
Assessors blinded	No	No	No	No	No	No	No	No	No	No	No	No
Loss to follow-up <20%	No	CD	CD	CD	CD	CD	CD	CD	No	CD	CD	CD
Key potential confounders measured	Yes	CD	No	No	No	Yes	No	CD	No	CD	Yes	No

*CD* could not determine, *NR* not reported, *NA* not applicable

**Table 3:** Quality assessment before–after studies

	Bumm et al.^18^	Dickinson et al.^20^	Freeman et al.^25^	Pawlik et al.^22^
Objective clearly stated	Yes	Yes	No	Yes
Eligibility criteria specified	Yes	Yes	Yes	Yes
Participants’ representative	Yes	Yes	Yes	Yes
All eligible participants enrolled	Yes	Yes	Yes	Yes
Sample size sufficiently large	NR	NR	NR	NR
Intervention clearly described	Yes	Yes	Yes	Yes
Outcome measures specified	No	Yes	No	Yes
Assessors blinded	No	No	No	No
Follow-up	CD	CD	CD	NR
Statistical methods	NA	Yes	Yes	NA
Outcome measures taken multiple times before and after intervention	No	No	No	No
Statistical analysis for group to individual effect	NA	NA	NA	NA

*CD* could not determine, *NR* not reported, *NA* not applicable

**Table 4:** Risk of bias and quality of included studies

Bias assessed with the Cochrane tool to “Assess risk of bias in cohort studies” (Appendix). Quality assessed with the quality assessment tool for “Before–after studies” and “For observational cohort and cross-sectional studies” from the National Heart, Lung, and Blood Institute (Appendix)

*1* indicates ‘definitely yes’ (low risk of bias), *2* indicates ‘probably yes’, *3* indicates ‘probably no’, *4* indicates ‘definitely no’ (high risk of bias)

### Diagnoses and Staging

Eight studies described diagnoses for patients with GI malignancies, formulated by an MDT.^13^,^22^,^24^,^25^,^27^,^30^^–^ 32 The study by Basta et al. described whether changes in the initial diagnosis or stage were validated with either pathology or follow-up.^31^ In this study (various GI malignancies, *n* = 550), no diagnosis or stage was changed after validation by pathology or after follow-up.^31^ Six studies investigated diagnoses formulated by the MDT, of which five described that a different diagnosis was formulated by the MDT compared with the diagnosis formulated by the referring physician.^22^,^24^,^30^^–^ 32 Four of these studies found that 18.4–22.2% of the diagnoses were changed.^22^,^24^,^31^,^32^ Three studies regarding various GI malignancies (*n* = 551), pancreatic cancer (*n* = 203), and liver cancer (*n* = 343) described a proportion of patients who eventually had a benign diagnosis (6.0, 10.5, and 30.8%, respectively). The study by Meguid et al. (various GI malignancies, *n* = 1747) did not describe whether a proportion of patients received a benign diagnosis after evaluation by the MDT.^32^ Furthermore, the study by Fernando and colleagues (colon cancer, *n* = 459) investigated these changes in a subset (*n* = 456/459) of their patient population. They described that in only 4% (*n* = 20) of patients discussed, the clinical stage was changed.^30^

The four other studies regarding diagnoses formulated by the MDT described whether the diagnosis formulated by the MDT was correct.^13^,^25^,^27^,^31^ In the study by Davies et al. (gastric or esophageal cancer, *n* = 118), it was demonstrated that in 89% of patients discussed in an MDTM, a correct diagnosis was formulated.^13^ Basta and colleagues found that the MDT accurately diagnosed 93.5% of evaluated cases.^31^ In both studies, the diagnoses were validated by pathology or follow-up. The two other studies described the extent to which the discussion during the MDTM influenced staging.^25^,^27^ The study by Ye et al. (colorectal carcinoma, *n* = 595) found that after introduction of the MDTM, more patients underwent computed tomography (CT) examination before operation (55.7 vs. 30.0%). Ye et al. found fewer liver metastases in the post-MDT group, 6 months after resection,^27^ while the study by Freeman et al. (esophageal cancer, *n* = 255) found that patients discussed after the implementation of the MDTMs more often received a complete staging evaluation (97 vs. 67%).^25^ Complete staging evaluation was defined as “a minimum of CT and PET scans, esophagogastroduodenoscopy, bronchoscopy, complete blood count, electrolyte profile, and endoscopic esophageal ultrasonography, biopsy confirmation of suspected metastatic disease and further evaluation of any specific symptoms”.^25^

### Treatment Plan

Thirteen studies described treatment plans formulated by an MDT,^18^^–^ 25,28 ^–^ 32 nine of which described whether the treatment plan formulated by the referring physician was changed after discussion by the MDT.^18^,^21^^–^ 24,28 ^–^ 30,32 These studies reported on 42–1747 patients and found that 23.0–41.7% of the treatment plans formulated by the referring physician were changed after discussion by the MDT.^18^,^21^^–^ 24,28 ^–^ 30 Changes in the treatment plan could be divided into minor and major changes: minor changes were defined as additions to the originally stated treatment plan and occurred in 28.0–58.0% of the alterations made by the MDT; major changes encompassed an alteration in treatment modality and occurred in 41.5–72.0% of the changes formulated by the MDTs.^18^,^21^,^22^ Treatment plans were most often changed after an alteration of the initial diagnosis or stage.^22^,^24^,^28^ In the study by Pawlik et al., 77% of changes in treatment were led by a change in diagnosis or stage, while Snelgrove and colleagues found that 100% of changes in treatment were due to changes in diagnosis.^22^,^28^ In the study by Zhang et al., only 33% of changes in treatment were due to a change in diagnosis or stage.^24^ In six studies the authors did not report why the initial treatment plan formulated by the referring physician was changed after discussion at an MDTM.^18^,^21^,^23^,^29^,^30^,^32^

The three remaining studies focused on adherence to guidelines by an MDT^19^,^20^,^25^; Dickinson et al. (pancreatic body cancer, *n* = 31), observed that more patients received chemotherapy after being discussed at an MDTM (43 vs. 25%),^20^ while Burton et al. (rectal cancer, *n* = 298) used the circumferential resection margin (CRM) as an indicator for the quality of their MDT.^19^ After implementation of mandatory preoperative magnetic resonance imaging (MRI) and discussion at the MDTM, there were significantly less positive CRMs: 1% after implementation of the MDTM versus 26% before implementation.^19^ Patients not discussed at the MDTM did not receive preoperative neoadjuvant treatment. In the third study, Freeman et al. (esophageal cancer, *n* = 255) found that for patients discussed at an MDTM, the treatment plan more often adhered to national guidelines: 98 versus 83%.^25^

### Implementation of the Treatment Plan

Two articles on patients with colorectal carcinoma (*n* = 185 and 201), two articles on esophageal and gastric cancer (*n* = 42 and 2450), and one article on various GI malignancies (*n* = 551) analyzed whether treatment decisions formulated by the MDT were actually implemented.^18^,^26^,^28^,^29^,^31^ The implementation rate ranged from 90 to 100%. The reasons for not following MDT advice were comorbidity (45%) and patient preferences (35%), followed by new clinical information (10%), different opinion of the treating physician (5%), and unknown (5%).^26^,^29^

## Discussion

This systematic review focuses on the changes in diagnosis and treatment plan formulated at the MDTM for patients with a GI malignancy. Changes in diagnosis occurred for 18.4–26.9% of the evaluated patients, and changes in treatment occurred in 23.0–41.7% of the evaluated patients. To our knowledge, this is the first systematic review to assess these outcomes in the field of GI oncology.

Discussion of patients in an MDTM has many advantages. Different medical specialists convene to discuss diagnoses, view radiographic imaging, and review pathology. Additionally, an MDTM facilitates exchanging knowledge and ensures a more extensive understanding regarding the treatment possibilities of other medical specialties. Both diagnostic capabilities and therapeutic options can be easily discussed to ensure the best treatment for each individual patient.

For all patients, it is important to receive a correct diagnosis and staging of the disease since this will lead to a correct treatment plan. Patients discussed at an MDTM seem to have more accurate diagnoses than patients diagnosed by a single physician. This statement is supported by the observed changes made in diagnoses for patients discussed in an MDTM (18.4–26.9%). It is probable that team members better adhere to diagnostic protocols and therefore formulate more accurate diagnoses.^25^,^27^ Although the included studies show little evidence as to whether these changes in diagnoses are accurate, both Basta et al. and Davies et al. proved that diagnoses formulated at an MDTM are often correct.^13^,^31^

We also found that discussion at an MDTM can lead to a different treatment plan that better adheres to existing guidelines; however, none of the authors of the included studies described which guidelines were used at their institute. Treatment plans for patients with a GI malignancy formulated by an MDT are often implemented. The major reasons for not implementing a treatment plan included patient morbidity and patient preferences; it is important to know the patient’s condition and wishes in advance. There are several ways to ensure the patient’s wishes and physical condition are taken into account. The presence of a physician who has met the patient is one the most influencing factors to ensure that due attention is paid to this.^31^ Other ways of ensuring this also exist, i.e., nurses, nurse practitioners, or psycho-oncologists can be employed to ensure these aspects are incorporated into the decision-making process. Additionally, the patient can be present at the MDTM to ensure his wishes are taken into account when formulating a treatment plan; however, the latter is not often employed, which could be due to several reasons. During MDTMs, medical terminology is often used. For patients, this is generally difficult to understand and could even be perceived as frightening.^33^ It is possible that patients are not yet aware of their diagnosis at the time of the MDTM. It is of course unacceptable to tell patients their diagnosis within the 4 min timeframe usually used to discuss patients during an MDT.^31^,^33^^–^ 35 Additionally, it seems most healthcare providers do not wish to have patients present during the MDTM.^35^

One of the strengths of this review is renewed attention to a subject that has become integrated in our healthcare system. Our systematic review corroborates the conception that MDTMs positively contribute to modern healthcare. This review also has some limitations. The group of patients with GI malignancies is still fairly heterogeneous, therefore a quantitative analysis, such as a meta-analysis, was not possible. The types of studies included in this review have all used study methodologies more prone to bias and of lesser scientific quality, i.e. (retrospective) cohort studies or before–after studies. This is common in comparative studies assessing healthcare outcomes.^6^,^11^,^36^,^37^ We have found several definitions used to describe MDTMs, however due to the wide variability in the definitions used in the literature, it is possible there are definitions unknown to us. The different definitions used for MDTs, as well as the differences in outcome measures, further increase the difficulty of objectively studying the effect of MDTs and MDTMs on healthcare; it is possible relevant literature has been missed. No studies reported on an MDT performing worse than a single physician, although one study reported no difference,^25^ which could mean MDTs actually ensure enhanced care is delivered, or a publication bias was evident. In most studies, whether a patient was discussed by an MDT was at the sole discretion of the treating physician. Both of these can cause discrepancies between the pre- and post-MDT groups. None of the studies have taken into account changes in treatment over time.

## Conclusions

The majority of the reviewed studies found that MDTs have definite advantages: better adherence to guidelines, better diagnostics, and better adherence to formulated treatment plans. These advantages seem to outweigh the disadvantages, i.e., economic costs. To objectively compare the advantages with the disadvantages, MDTs and MDTMs should be more clearly defined. Additionally, their outcome measures should also be defined. Studies and reviews that show how MDTMs influence patient care and work satisfaction are of the utmost importance. However, in light of the presented evidence, we firmly believe in both the benefit and durability of MDTMs; MDTs are here to stay!

## Electronic supplementary material

Below is the link to the electronic supplementary material.

Supplementary material 1 (DOCX 16 kb)
