# Supplementary material for: The Value of Multidisciplinary Team Meetings for Patients with Gastrointestinal Malignancies: A Systematic Review
Source: Ann Surg Oncol. 2017 Mar 23;24(9):2669–78. doi: 10.1245/s10434-017-5833-3 (PMC5539280; doi:10.1245/s10434-017-5833-3)
Supplement: Supplementary file 1 — Supplementary material 1 (DOCX 16 kb) [file 10434_2017_5833_MOESM1_ESM.docx]

**Medline**

(exp "Delivery of Health Care, Integrated"/ or interdisciplinary communication/ or exp Patient Care Team/ or (cancer conference* or MULTIDISCIPLIN* or interdisciplinar* or ((Centre* or center*) adj3 excellen*) or (integrated adj3 care) or (care adj2 team) or (tumo?r adj2 board)).ti,ab,kw.) and (exp Early Diagnosis/ or exp Diagnostic Errors/ or exp Neoplasm Staging/ or Palliative Care/ or exp Guideline Adherence/ or exp "Drug-Related Side Effects and Adverse Reactions"/ or exp Disease-Free Survival/ or exp Interrupted Time Series Analysis/ or exp Controlled Before-After Studies/ or ae.fs. or (((alternative or alteration or issue* or modif* or vary or improve or improving or chang*) adj5 (staging or treatment or stage or diagnosis)).ti,ab. or adverse event*.ti,ab,kw. or ((palliative or better or optimal) adj3 care).ti,ab,kw. or (cluster* adj2 random*).ti,ab,kw. or (before adj after).ti,ab,kw. or interrupted time ser*.ti,ab,kw.))

| exp Digestive System Neoplasms/ |
| --- |
| 1 and 2 |
| case reports.pt. |
| 3 not 4 |
| review.pt. |
| 5 not 6 |

**Embase**

(exp *"integrated health care system"/ or *interdisciplinary communication/ or *multidisciplinary team management/ or *teamwork/ or exp *patient care/ or (cancer conference* or MULTIDISCIPLIN* or interdisciplinar* or ((Centre* or center*) adj3 excellen*) or (integrated adj3 care) or (care adj2 team) or (tumo?r adj2 board)).ti,ab,kw.) and (exp early diagnosis/ or exp diagnostic error/ or exp cancer staging/ or palliative therapy/ or exp practice guideline/ or exp "adverse drug reaction"/ or exp side effect/ or exp "disease free Survival"/ or exp time series analysis/ or ae.fs. or (((alternative or alteration or issue* or modif* or vary or improve or improving or chang*) adj5 (staging or treatment or stage or diagnosis)).ti,ab. or adverse event*.ti,ab,kw. or ((palliative or better or optimal) adj3 care).ti,ab,kw. or (cluster* adj2 random*).ti,ab,kw. or (before adj after).ti,ab,kw. or interrupted time ser*.ti,ab,kw.))

| exp digestive system cancer/ |
| --- |
| 1 and 2 |
| "review"/ |
| case report/ |
| limit 3 to (conference abstract or conference paper or conference proceeding or "conference review") |
| 4 or 5 or 6 |
| 3 not 7 |

**Pubmed**

Search ((cancer conference*[tiab] or MULTIDISCIPLIN*[tiab] or interdisciplinar*[tiab] or ((Centre*[tiab] or center*[tiab]) AND excellen*[tiab]) or integrated care[tiab] or care team[tiab] or tumor board[tiab] or tumour board[tiab]) and (((alternative[tiab] or alteration[tiab] or issue*[tiab] or modif*[tiab] or vary[tiab] or improve[tiab] or improving[tiab] or chang*[tiab]) AND (staging[tiab] or treatment[tiab] or stage[tiab] or diagnosis[tiab)) or adverse event*[tiab] or ((palliative[tiab] or better[tiab] or optimal[tiab]) AND care[tiab]) or (cluster*[tiab] and random*[tiab]) or "before and after"[tiab] or interrupted time ser*[tiab])) AND publisher[sb] Filters: Publication date from 2015/01/01 to 2030/12/31
